# Artificial intelligence-driven clinical auxiliary diagnosis of benign paroxysmal positional vertigo

**DOI:** 10.3389/fneur.2026.1774729

**Published:** 2026-02-17

**Authors:** Siyang Dai, Ying Wu, Xiaocui Kang, Zuoting Shen, Ping Zhong

**Affiliations:** Department of Neurology, Shidong Hospital Affiliated to University of Shanghai for Science and Technology, Shanghai, China

**Keywords:** artificial intelligence, benign paroxysmal positional vertigo, deep learning, machine learning, nystagmus, vertigo

## Abstract

Benign paroxysmal positional vertigo (BPPV) is one of the most prevalent peripheral vertigo disorders in clinical practice. Its definitive diagnosis relies heavily on characteristic nystagmus induced by positional provocative tests, which imposes high requirements on clinicians and is subject to limitations such as strong subjectivity. The breakthrough advances in artificial intelligence (AI) technologies have provided innovative solutions for the accurate diagnosis and personalized treatment of BPPV. This review systematically summarizes the research progress of AI in the clinical application of BPPV, its enormous potential to improve BPPV diagnostic efficacy, and future directions for development.

## 1 Introduction

Episodes of BPPV are mostly associated with specific changes in head position, with common precipitating factors including movements such as getting in and out of bed, turning over in bed, tilting the head backward, and bending it forward; its core clinical feature is paroxysmal and transient vertigo that typically lasts no more than 1 min, and its pathogenic mechanism is directly linked to changes in the position of the head relative to the gravitational field. The widely accepted pathological mechanism of BPPV is that calcium carbonate otoconial crystals detach from the utricle of the inner ear, then float freely in the lumen of the semicircular canals or adhere to the wall of the ampulla, leading to abnormally increased sensitivity of the vestibular labyrinth to gravitational stimuli (1). As the most prevalent peripheral vertigo in clinical practice, BPPV accounts for approximately 20–30% of all patients with vertigo, with the peak age of onset occurring around 60 years; in addition, this disorder has a relatively high recurrence tendency, with an annual recurrence rate of about 15–20% (2). In clinical diagnosis and treatment, the posterior semicircular canal is the most commonly affected site in BPPV cases, accounting for roughly 85% of all instances. The horizontal semicircular canal is involved in approximately 15% of cases, whereas involvement of the anterior semicircular canal is the rarest, with an incidence rate of only about 1% (3–5).

Most middle-aged and elderly patients have comorbid chronic conditions such as hypertension and diabetes mellitus, which complicates the diagnosis of BPPV; such misdiagnosis frequently leads to the implementation of unnecessary diagnostic procedures, patient referrals, and therapeutic interventions. If not diagnosed and treated promptly, BPPV can lead to a decline in quality of life and an increased risk of falls, which are the leading cause of hospitalization among the elderly due to injuries and trauma (6).

**Figure 1:**
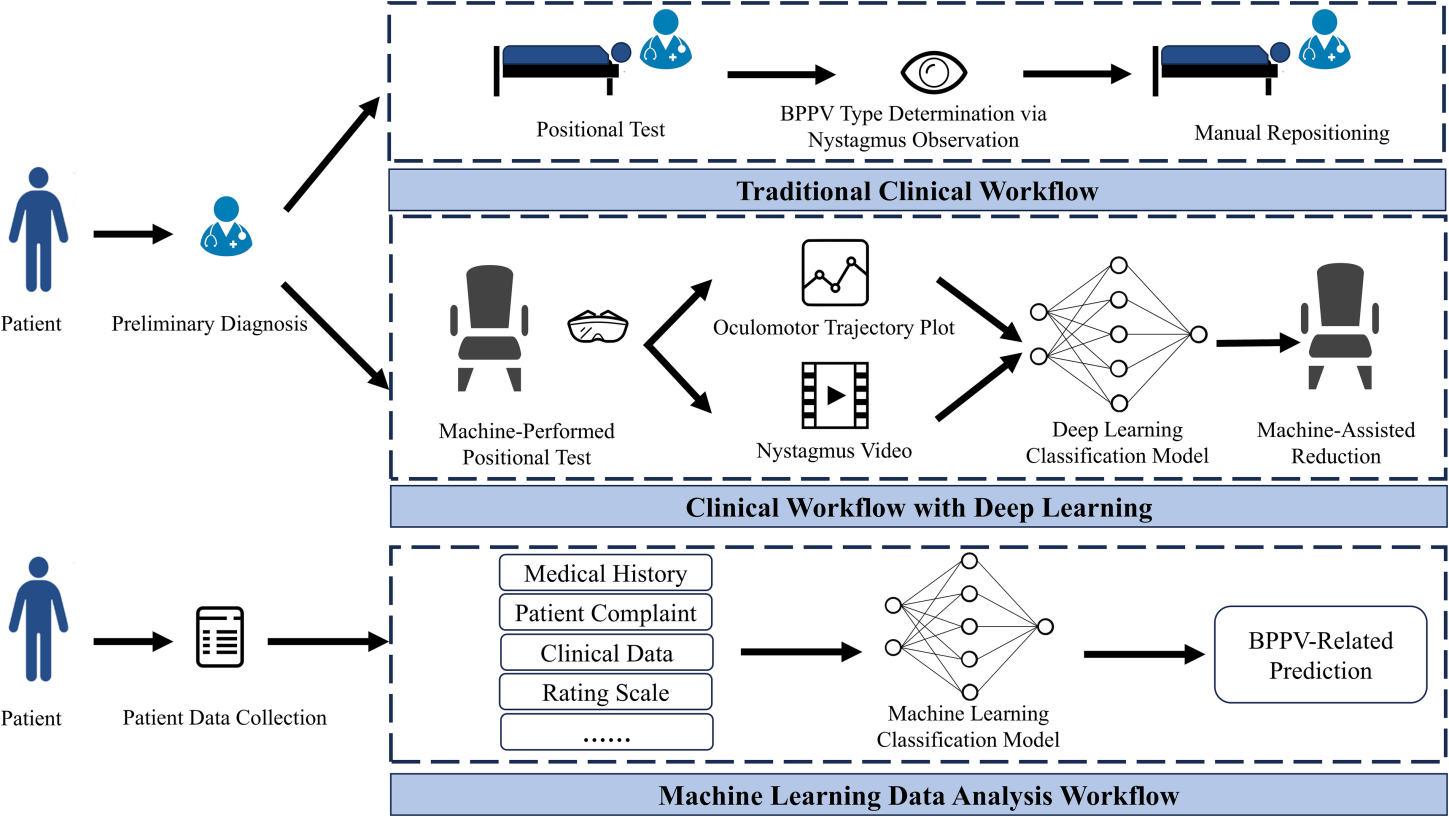
Workflow diagrams of different methods.

In the traditional diagnostic and treatment model for BPPV, clinicians can identify the affected semicircular canal by having patients adopt different body positions (Dix-Hallpike test and Roll test), observing their eye movements, and asking whether they experience vertigo symptoms, after which targeted repositioning therapy is administered. However, this diagnostic and treatment pathway has certain limitations: the procedure is time-consuming and laborious for patients with obesity or cervical spine disorders; patients may be uncooperative and unable to keep their eyes open, and the approach places high demands on clinicians’ clinical experience and operational skills, rendering it highly susceptible to subjective factors. AI technology, therefore, offers a vital direction for innovation in this field (7–9). Figure 1 shows a schematic diagram of the clinical workflows for different diagnostic and treatment methods.

This review focuses on the application methods of AI in the auxiliary diagnosis of BPPV, systematically summarizes the relevant research progress, thoroughly discusses the specific challenges faced by the application of neural network technology in this field and the corresponding solutions, and analyzes the limitations of current research as well as future development directions.

## 2 Methods

This systematic review was conducted in accordance with the PRISMA guidelines to ensure transparency, reproducibility, and methodological rigor. A literature search was performed across the PubMed, Web of Science, and Google Scholar databases for articles published between January 2015 and June 2025. The search strategy for this study centered on key terms: artificial intelligence, benign paroxysmal positional vertigo, nystagmus, and vertigo; these terms were combined via Boolean operators (AND, OR) to ensure comprehensive coverage of relevant literature.

**Figure 2:**
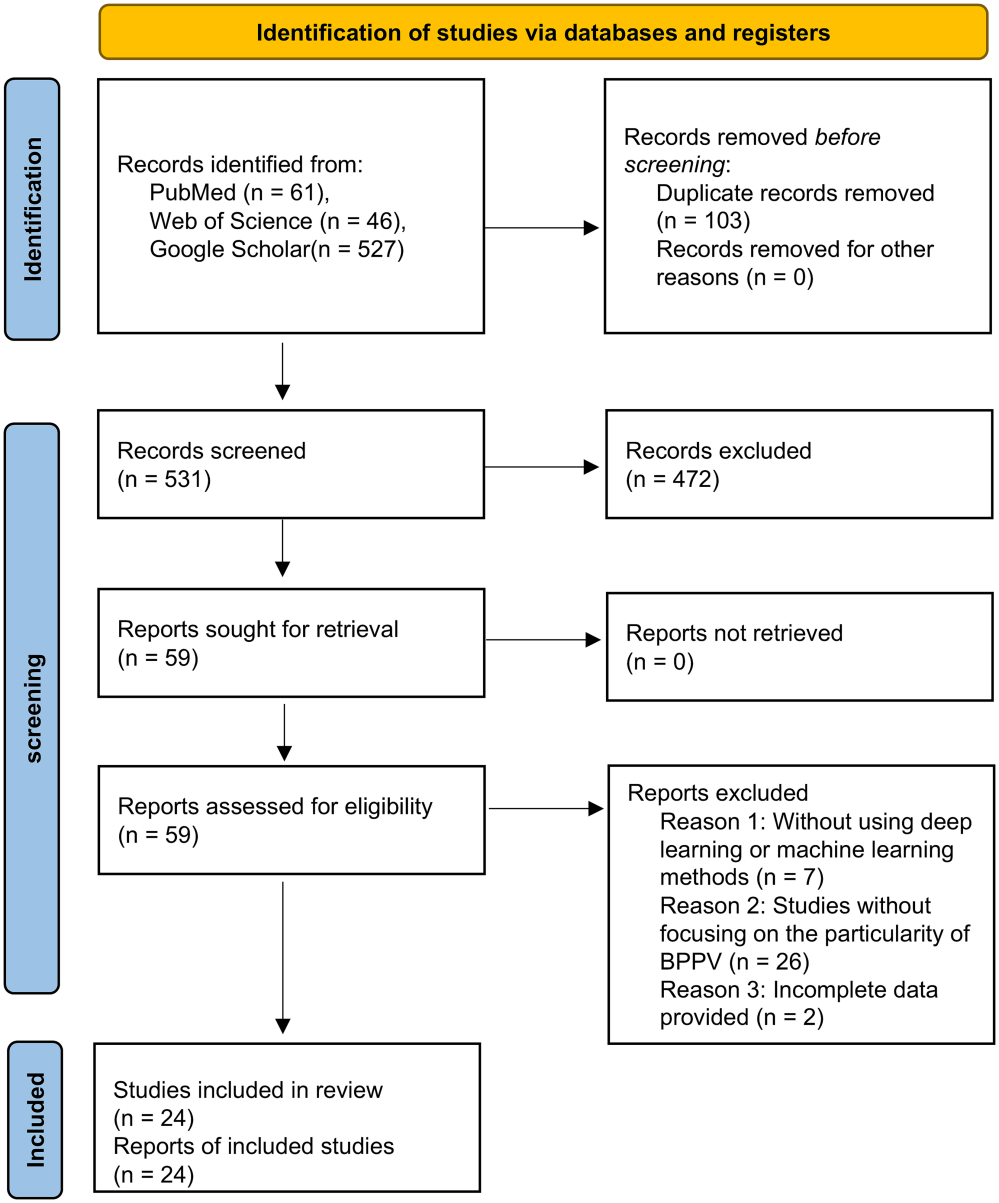
PRISMA flow diagram.

Initial retrieval using this strategy yielded a total of 634 articles. After removing duplicate entries, 531 articles remained for further evaluation. A preliminary screening based on titles and abstracts excluded 472 articles, leaving 59 studies for full-text review. Following a detailed assessment, 35 additional articles were excluded on the basis of eligibility criteria. Ultimately, 24 articles that met all inclusion standards were included in the final analysis. The study selection process is illustrated in Figure 2, following the PRISMA flow diagram.

## 3 Fundamentals of artificial intelligence technology

Machine learning is one of the core technologies of artificial intelligence, among which supervised learning and unsupervised learning are the most widely adopted. Supervised learning relies on labeled training data to learn the mapping relationship between inputs and outputs, enabling the prediction of new data, and is mostly applied to solve classification or regression problems. In contrast, unsupervised learning only uses unlabeled data to drive models to autonomously mine hidden structures and inherent patterns in data, and is usually employed for clustering or dimensionality reduction tasks (10).

As an important branch of machine learning, deep learning takes multi-layer neural networks as its core architecture and can automatically extract high-dimensional features and identify complex data patterns from massive datasets (11). Convolutional Neural Networks possess excellent autonomous learning and feature representation capabilities; after training, they can complete automatic feature extraction from raw input data without human supervision. By comparison, Recurrent Neural Networks leverage cyclic structural units to effectively process sequential data, capturing sequential dependencies in data by transmitting time-series information across different moments (12, 13).

Machine learning provides basic support for data pattern mining through differentiated data processing modes, while deep learning enhances the advantages of complex feature extraction and sequential data processing via specific network structures. These technical characteristics lay a solid foundation for the application of artificial intelligence in scenarios such as feature analysis, mechanism exploration, and prognosis prediction for the clinical auxiliary diagnosis of BPPV (14).

## 4 Application of artificial intelligence in nystagmus analysis

### 4.1 Eye tracking

Nystagmus refers to the involuntary, rhythmic, reciprocating movement of the eyes when fixing gaze on a specific point. Based on the direction of this rhythmic oscillation, nystagmus can be classified into horizontal nystagmus, vertical nystagmus, and torsional nystagmus. In clinical diagnosis, the core diagnostic criterion for BPPV is the nystagmus manifestations observed during a patient’s vertigo episode. The types of nystagmus induced by BPPV mainly include horizontal nystagmus and vertical upbeat nystagmus with a torsional component. Currently, the standard clinical tool for data acquisition is the head-mounted video oculography system. This device incorporates infrared cameras, infrared light-emitting diodes, and goggle assemblies to enable accurate capture of dynamic eye movement videos. In AI-assisted BPPV diagnostic research, the accurate localization of the ocular region serves as an indispensable core prerequisite and fundamental step.

Rodrigues et al. (15) proposed the At-UNet neural network model, which adopts VGG16 as its backbone encoder. By integrating an attention module and a multi-task learning framework, the model achieves simultaneous and accurate segmentation of the pupillary region, yielding a Dice coefficient of 96.20% for pupil segmentation on the UTIRIS dataset. However, the model suffers from a large number of parameters, which hinders its lightweight deployment in clinical settings. Wei et al. (16) combined the YOLOv5 object detection network with an improved DeepLabv3 + segmentation module; precise pupillary coordinates were obtained via ellipse fitting of segmentation masks, with the final intersection over union (IoU) reaching 95.95%. While this approach is well-suited for real-time clinical requirements, the ellipse fitting method exhibits poor adaptability to pathologically irregular pupils and is thus prone to generating deviations.

Ideal acquisition of patients’ ocular information is often hindered by various interferences. Cho et al. (17) developed a lightweight multi-task model that integrates a blink detection module into the pipeline of pupil localization and tracking, which specifically addresses invalid frames caused by eye blinking; the model achieved IoU values of 92.81 and 90.73% on the OpenEDS and HUSHH datasets, respectively. While well-suited for dynamic clinical scenarios and characterized by low deployment barriers, this multi-task architecture leads to slightly reduced precision in individual tasks, and its sensitivity to the recognition of rapid consecutive eye blinks requires further improvement. To evaluate the performance of pupil segmentation algorithms under different noise conditions, Ju-Hyuck et al. (18) proposed a combined RANSAC+U-Net scheme. Results showed that the RANSAC+U-Net combined algorithm performed optimally in the noise-free scenario with a mean squared error (MSE) of 0.0620; the standalone U-Net algorithm excelled in the optical noise scenario with an MSE of 0.0694; and the standalone RANSAC algorithm yielded the best performance in the motion blur noise scenario with an MSE of 0.0717. However, all algorithms exhibited poor performance in the presence of human-induced noise, such as occlusion by eyelids and eyelashes. The core underlying reason is the lack of critical image features, coupled with the absence of effective feature completion mechanisms in existing models, which renders them unable to accommodate the variability of complex physiological structures.

Choi et al. (19) proposed an automatic eyeglass removal method based on the CycleGAN network. The primary objective of a generative adversarial network is to use a discriminator to calculate the distribution of original samples, while a generator works to generate new samples from real data samples (20). This method is applied to supplement the key information of the ocular region that is lost due to the presence of eyeglasses. In future research, we can also explore solutions to problems such as data loss and noise interference in pupil localization by leveraging generative adversarial networks.

In summary, compared with the iris, the pupil offers the advantages of high stability, strong anti-interference capability, and low algorithm complexity, making it more suitable for meeting the technical requirements of nystagmus video analysis and aligning with the core clinical diagnostic demands of speed, real-time performance, and anti-interference capability. Although artificial intelligence is currently capable of pupil localization and tracking, there remains significant room for optimization in numerous aspects.

### 4.2 Application of ocular movement trajectory

The ocular movement trajectory plot converts subjectively observable nystagmus into objectively quantifiable trajectory curves and data, thus avoiding deviations caused by manual judgment. To address the challenge of accurate pupil tracking, Lee et al. (21) developed ANyEye, an AI-assisted nystagmus video analysis system that integrates a compensation algorithm to correct pupil positions, achieving a detection rate of 91.26% for pupil tracking within a 5-pixel error margin, making it well-suited for dynamic clinical tracking applications. However, its adaptability to high-velocity nystagmus scenarios was not reported in the study. Deng et al. (22) proposed the lower pole of pupil algorithm and employed ResNet34 for classifying four common subtypes, with the accuracy rate reaching 95.55%. Regarding the issue of data loss caused by pupil occlusion due to various factors, Mun et al. (23) pointed out that linear interpolation, if adopted as a missing data bridging algorithm, might inadvertently generate nystagmus-like motion artifacts; filling missing values with the pupil position detected at the previous moment (denoted as NA) yields better results. Ultimately, the CNN1D model was used, achieving an accuracy rate of 91.02%. This approach provides a quantitative reference for missing data handling, though its effectiveness in scenarios involving prolonged pupil occlusion remains to be verified.

Due to the limited information that can be conveyed by a single trajectory plot, many researchers have begun to explore converting trajectory plots into other forms of information for nystagmus identification. Dogru et al. (24) transformed the original trajectories into polar coordinates and calculated angular changes via template matching, successfully addressing the challenge of torsional nystagmus detection. Qiu et al. (25) completed classification after converting trajectories into Gram matrix feature images, achieving a Top-1 accuracy of 85.47%. Lee et al. (26) utilized the wavelet transform to convert time-series signals into time-frequency images, which were ultimately fed into the EfficientNet convolutional neural network for classification, yielding an overall accuracy of 87%. Although different data conversion strategies can improve the accuracy of nystagmus identification from specific dimensions, such transformation processes are often associated with several limitations. These drawbacks include the easy loss of temporal information during feature mapping and the lack of unified criteria for wavelet transform parameter settings, which exert a notable impact on the final results.

In addition, the adoption of a multimodal approach that incorporates more clinical information for analysis can improve diagnostic accuracy. Wu et al. (27) converted eight features, including head trajectory, eye movement trajectory, and their corresponding slow-phase velocity values, into 1D data as input. Nguyen et al. (28) fused five-channel time-series data consisting of horizontal eye movement, vertical eye movement, pupil radius, horizontal velocity, and vertical velocity. Going beyond nystagmus-related information alone, Liu et al. (29) adopted a multi-technology fusion strategy combining image features and signal analysis to conduct comprehensive nystagmus detection. Although such multimodal fusion methods can significantly enhance the generalization ability of models in clinical settings, they inevitably increase model complexity, thereby raising the bar for clinical deployment and implementation.

In clinical practice, physicians still rely primarily on the direct interpretation of eye movement images as the main diagnostic basis, and ocular movement trajectory plots have not yet become routine core diagnostic tools. Notably, current intelligent classification research on BPPV based on ocular movement trajectories has demonstrated significant clinical effectiveness and promising application prospects.

### 4.3 Application of nystagmus videos

With the gradual emergence of intelligent video analysis technology as a research hotspot in the field, relevant research directions have also begun to focus on nystagmus video analysis, which serves as the core carrier for clinical diagnosis. Li et al. (30, 31) designed different deep learning algorithms integrating multiple modules for vertical nystagmus and torsional nystagmus, achieving an accuracy of 91 and 96.1%, respectively. However, the adaptability of these algorithms to complex clinical scenarios in practical experiments remains to be verified. Lim et al. (32) developed a 2D-CNN model that converts the 3D eye movement features in videos into grid images for classification. Results showed that the area under the curve (AUC) for horizontal nystagmus and vertical nystagmus reached 0.966 and 0.952, respectively, while the AUC for torsional nystagmus was only 0.853. The main limitation lies in the fact that the identification of torsional nystagmus relies on the accurate capture of iris rotation states. In clinical infrared videos, low brightness and contrast often blur iris textures, impeding feature extraction.

To address the problem of limited recognition accuracy for torsional nystagmus, researchers have introduced optical flow technology. The core principle of optical flow is to estimate pixel displacement between consecutive video frames for accurate capture of motion dynamics (33). Kong et al. (34) used LiteFlowNet to extract optical flow features, which were then fused and classified via the nystagmus video classification network based on temporal modeling. This method achieved an F1-score of 0.98 for torsional nystagmus, surpassing the 0.928 score obtained for non-torsional nystagmus. Zhang et al. (35) proposed a Torsion-aware Bi-Stream Identification Network, which inputs optical flow in the x and y directions into the two-stream network for torsional nystagmus recognition, reaching an accuracy of 85.73% in clinical evaluations. Model designs incorporating optical flow fields are more compatible with the characteristics of clinical videos, effectively resolving the recognition challenges caused by blurred iris textures. Nevertheless, optical flow feature extraction imposes certain computational requirements, which may increase the costs associated with clinical deployment.

In addition to optical flow features, conducting multimodal fusion research that incorporates the multidimensional clinical characteristics of vertigo associated with BPPV can also effectively improve the diagnostic accuracy of the model. Lu et al. (36) encoded head position vectors using an autoencoder to capture spatial information, and fused the encoded information with video features via a cross-attention mechanism, achieving an average accuracy of 81.7%. While this approach enabled the synergistic utilization of head posture and eye movement information, it suffered from the drawback of high computational complexity during the feature fusion process. Pham et al. (37) developed a hybrid deep learning system named “Look and Diagnose”, which integrates body posture and binocular vision information. The system first detects posterior semicircular canal BPPV and then classifies non-posterior semicircular canal otolithiasis, with an overall classification accuracy of 91% and demonstrating strong alignment with clinical diagnostic workflows. Table 1 systematically presents the research on deep learning related to nystagmus video analysis. These studies indicate that deep learning has currently achieved favorable results in the clinical image analysis of BPPV and can provide references for clinical practice. Nevertheless, continuous optimizations are still required in terms of computational cost control, generalization capability in complex scenarios, and adaptability to clinical workflows.

**Table 1:** Summary of deep learning research related to nystagmus analysis.

Researchers	Data set	Model	Performance
Rodrigues et al. (2024) (15)	433 videos	At-Unet+Attention	Dice similarity coefficient: 96.2%
Wei et al. (2022) (16)	TEyeD	YOLOv5-DeepLabv3+	IOU: 95.95%
Cho et al. (2024) (17)	HUSHH+OpenEDS	Lightweight model	IOU: 90.73% (HUSHH), 92.81% (OpenEDS)
Ju-Hyuck et al. (2025) (18)	CASIA-Iris-Degradation	ANSAC+U-Net	MSE: 0.0620
Lee et al. (2023) (21)	52 patients	ANyEye	Detection rate at 5-pixel error: 91.26%
Deng et al. (2023) (22)	433 nystagmus videos	ResNet34	Accuracy: 95.55%
Mun et al. (2024) (23)	828 patients	2D U-Net + CNN1D	Accuracy: 91.02 ± 0.66%
Qiu et al. (2023) (25)	646 VNG videos	Gram-AODE	Top-1 accuracy: 85.47%
Lee et al. (2024) (26)	947 VNG videos	EfficientNet	Accuracy: 87%
Wu et al. (2023) (27)	3,296 patients	1DCNN-BiLSTM-Self-attention	Accuracy: 93.3 ± 1.0%
Nguyen et al. (2025) (28)	LAD	EfficientNet-B0 + 1D CNN	Accuracy: 91%
Liu et al. (2025) (29)	60 patients	Egeunet	Accuracy: 93.33%
Li et al. (2023) (30)	21,743 videos	Bilstm−GRU module	Accuracy: 91%
Li et al. (2023) (31)	24,521 videos	Inception+BiLSTM	Accuracy: 96.1%
Lim et al. (2019) (32)	91,778 videos	2D-CNN	F1-score: 0.794 ± 0.008
Kong et al. (2023) (34)	728 videos	ConvNeXt+ LSTM+ Optical flow	F1-score: Non-torsional nystagmus: 0.928, Torsional nystagmus: 0.98
Zhang et al. (2021) (35)	77 videos	TSBIN	Accuracy: 85.73%
Lu et al. (2024) (36)	518 patients	BKTDN+ self-encoder+cross-attention	Accuracy: 81.7%
Pham et al. (2022) (37)	746 data from patients	“Look and Diagnose”	Accuracy: 91%

## 5 Application of artificial intelligence in clinical practice of BPPV

### 5.1 Diagnosis of BPPV

In clinical practice, physicians usually make a preliminary diagnosis of the disease by inquiring about the characteristics, triggers, and course of vertigo episodes, as well as the presence of accompanying symptoms or medical history. Accurately differentiating BPPV from other vestibular disorders requires a certain level of clinical experience on the part of physicians, which results in relatively high rates of missed diagnosis and misdiagnosis of BPPV in clinical practice.

In a machine learning study involving 7,660 patients, Khani et al. (38) allocated BPPV patients and non-BPPV controls at a ratio of 1:1. After preprocessing the demographic characteristics and clinical history features, they adopted multiple machine learning models to predict BPPV. The results showed that the gradient boosting model exhibited the best performance, with an accuracy rate of 85.422%. Compared with the approximately 70% misdiagnosis rate of peripheral vestibular disorders in emergency departments (39), this method achieved a significant improvement. Han et al. (40) established a multivariate logistic regression prediction model based on the clinical data and biomarkers of 522 patients, which achieved an AUC of 0.927.

Soylemez et al. (41) analyzed 280 patients with posterior semicircular canal BPPV. The results indicated that age, symptom onset time, symptom duration, dizziness type, triggering factors, and auditory symptom status were significant features. Using a random forest model, the diagnostic accuracy for posterior semicircular canal BPPV reached 96.43%.

### 5.2 Pathophysiological mechanism of BPPV

To date, the pathophysiological mechanism of BPPV has not been fully elucidated. Previous studies have suggested that it may be associated with factors such as hypertension and vitamin D deficiency (42). Based on a large-sample systematic analysis, Han et al. (40) used laboratory biomarker information as the core input—including routine blood test parameters, inflammatory and metabolic indicators, etc. The results indicated that disease course, neutrophil count, lymphocyte count, C-reactive protein, ferritin levels, and vitamin D deficiency were identified as independent risk factors for BPPV, while monocyte count was found to be a protective factor. These findings further suggest that inflammatory responses, iron metabolism disorders, and vitamin D deficiency may contribute to the development of BPPV.

### 5.3 Prognostic prediction of BPPV

There are significant individual differences in the clinical phenotypes and treatment outcomes of BPPV. Clinical data indicate that the proportion of patients requiring multiple canalith repositioning procedures can reach 36.6% (43). To accurately identify populations at high risk of multiple repositioning treatments and optimize clinical intervention strategies, Baydan-Aran et al. (44) retrospectively enrolled 520 BPPV patients. They selected clinical baseline data as input features, including age, gender, BPPV subtypes, and comorbidities (such as hypertension, diabetes mellitus, cervical disorders, and hearing loss). Nine machine learning prediction models were constructed and compared. The results showed that the gradient boosting machine model exhibited the optimal performance with an AUC of 0.788. Further analysis revealed that age, hypertension, and hearing loss were key influencing factors for multiple repositioning treatments, among which hypertension exerted the most significant impact. This study provides a quantitative tool for the clinical identification of patients at risk of requiring multiple repositioning procedures.

## 6 Discussion

As a common benign vertigo disorder, BPPV has relatively straightforward treatment methods. However, its clinical manifestations are easily confused with other vertigo-related conditions such as Meniere’s disease and vestibular neuritis, resulting in persistently high rates of missed diagnosis and misdiagnosis. Moreover, recurrent episodes can severely affect patients’ quality of daily life, thus deserving greater attention. AI technology has emerged as a core driving force for advancing the precise diagnosis and treatment of BPPV, demonstrating tremendous potential both as an adjunct in clinical diagnostic workflows and in predicting the prognosis of repositioning therapy outcomes. Nevertheless, several issues and challenges remain regarding the practical application of AI in clinical settings. In many previous studies, researchers prioritized data uniformity by discarding non-compliant or substandard data samples, which led to information loss and limited the generalization ability of the developed models. Othéguy et al. (45) developed an eye-tracking system based on scleral contact lenses. This system consists of electronic components and a camera integrated into a pair of glasses, which is remotely powered by two vertical cavity self-emitting lasers embedded in the scleral lenses. The device can also be safely used to monitor eye movements even when the eyelids are closed, but it is accompanied by problems related to device wearing comfort, safety, and hygiene. In future research, developing a convenient and safe method for detecting eye movements under interference will be a major challenge and key research direction.

In addition, to gain recognition from both physicians and patients in clinical practice, the interpretability and processing speed of AI (46), as well as how to balance these three aspects, are issues that need to be addressed. Standardization and popularization of AI-assisted diagnosis constitute a crucial next step for its broader clinical integration (47). With the improvement of research in various fields in the future, AI will be able to provide more comprehensive auxiliary diagnosis and treatment schemes for BPPV, enhancing both efficiency and accuracy.
